# Supplementary material for: Effects of the COVID-19 Restrictions on Eating Behaviour and Eating Disorder Symptomology in Female Adolescents
Source: Int J Environ Res Public Health. 2022 Jul 11;19(14):8480. doi: 10.3390/ijerph19148480 (PMC9325224; doi:10.3390/ijerph19148480)
Supplement: Supplementary file 1 [file ijerph-19-08480-s001.zip › ijerph-1761585-supplementary.pdf]

International Journal of
Environmental Research and Public Health

Supplementary Materials

Effects of the COVID-19 Restrictions on Eating Behaviour and Eating Disorder Symptomology in Female Adolescents

Table S1. Means and standard deviations (SDs) of absolute measures and percentual changes of CIES factors before vs. during confinement by group (all changes occurred in a positive direction, indicating an increase in symptoms) (AN: anorexia nervosa, SRPD: self-reported psychiatric diagnosis, F1: restrictive eating, F2: overeating, F3: anxiety and depressive symptoms, F4: emotion regulation problems).

	AN (N=19)		Depression (N=20)		SRPD (N=45)		Control (N=138)	
	Mean	SD	Mean	SD	Mean	SD	Mean	SD
**Absolute measures**								
F1 before	18.2	5.3	21.2	5.3	21.0	4.6	19.0	4.2
F1 during	28.4	5.1	24.1	5.9	24.0	5.6	21.2	4.9
F2 before	17.9	8.5	21.0	7.0	21.7	7.6	18.5	5.5
F2 during	19.4	8.3	24.4	8.9	25.4	8.9	21.4	7.0
F3 before	18.0	5.5	27.8	6.7	25.6	6.4	19.8	5.2
F3 during	28.7	8.0	33.9	7.8	32.0	5.8	25.4	6.7
F4 before	9.7	3.3	15.4	4.8	13.1	3.8	10.4	3.2
F4 during	14.3	4.7	18.4	4.0	15.7	4.0	13.0	4.2
**Percentual changes (%)**								
F1	61.01	29.92	15.90	25.29	17.25	29.29	13.41	24.83
F2	11.11	20.85	17.05	27.07	22.49	39.88	16.66	22.24
F3	63.28	35.71	25.20	27.36	30.56	32.70	30.29	27.27
F4	51.55	36.99	25.09	32.91	25.09	32.91	26.70	31.45

Table S2. Means and SDs of SATAQ scores by group (SATAQ: Sociocultural Attitudes Towards Appearance Questionnaire)

SATAQ	Group	N	Mean	SD
Pressure	AN	19	3.75	1.03
	Depression	20	3.37	1.38
	SRPD	44	3.34	1.06
	Control	134	2.68	1.14
Awareness	AN	19	3.56	1.10
	Depression	20	3.36	1.20
	SRPD	45	3.30	0.94
	Control	138	2.70	1.00
Internalization	AN	19	3.47	1.02
	Depression	20	3.19	1.17
	SRPD	45	3.31	1.00
	Control	138	2.69	0.90

Table S3. Percentual rates of responses on usage of each coping strategy, mean and SDs of number of strategies used and mean overall helpfulness score of coping strategies (1=not helpful, 2=a little helpful, 3=fairly helpful, 4=helpful, 5=very helpful) by group

	AN	Depression	SRPD	Control
**Strategy Used (%)**				
Yoga	31.6	10.0	46.7	34.1
Daily routine	89.5	75.0	84.4	89.9
Day planning	73.7	60.0	86.7	85.8
Mild physical exercise	94.7	50.0	80.0	85.5
Enjoyable activities	94.7	85.0	93.3	97.8
Relaxing time with the family	78.9	65.0	77.8	79.7
Playing with the family	94.7	40.0	77.8	82.6
Virtual social contact with friends	84.2	65.0	91.1	89.9
Meal planning	84.2	30.0	55.6	50.0
Learn what you never had time to	52.6	30.0	64.4	73.2
Virtual social contact with family	89.5	35.0	73.3	77.5
Meditation	26.3	20.0	44.4	34.8
Relaxation exercises	36.8	25.0	62.2	58.7
Positive situations diary	15.8	20.0	48.9	27.5
Mindfulness	42.1	35.0	51.1	42.0
Pleasant internet activities	57.9	70.0	71.1	70.3
Positive thinking	84.2	55.0	91.1	87.0
Diaphragmatic, deep breathing	21.2	25.0	51.1	27.5
(Self-) reinforcement	47.4	50.0	71.1	65.2
Cooking with the family	73.7	60.0	51.1	60.9
Interrupting thoughts/behaviours	36.8	60.0	57.8	27.5
**Mean number of strategies used (SD)**	13.1 (4.1)	10.1 (1.0)	14.3 (4.2)	13.5 (4.1)
**Overall helpfulness score (SD)**	2.0 (0.86)	1.4 (0.56)	2.1 (0.9)	2.3 (0.8)
